# P264: Infection control program to rural community hospital in India - a reality

**DOI:** 10.1186/2047-2994-2-S1-P264

**Published:** 2013-06-20

**Authors:** D Sureshkumar, R Gopalakrishnan, K AbdulGhafur, V Ramasubramanian

**Affiliations:** 1 Infectious Disease, Apollo Hospitals, Chennai, India

## Introduction

Organized infection control (IC) programs have been successful in reducing the hospital-acquired infections (HAI). However, in India well organized IC programmes are confined to few corporate hospitals in urban areas. Rural community hospitals, although contributing significantly to the health care system, have none or inadequate IC programmes.

## Objectives

We here report the successful implementation of infection control program to a rural hospital for the first time in India.

## Methods

As a part of novel imitative to provide IC program to a 50 bed rural community hospital, infectious disease physician from the urban hospital visited the hospital periodically & through tele-conferences assessed baseline status & implemented the infection control program.

## Results

At baseline (October 2012) there was only two member infection control committee on paper without regular meetings, hand hygiene faculties were at the end of each ward with soap and water facility and compliance rate of 10%, no education program regarding infection control & prevention was conducted, and no HAI surveillance was done and finally no antibiotic stewardship program was present in the hospital. In the next 6 months (March 2013) with the infection control implementation the IC team expanded to include nurse, infectious disease physician and housekeeping staff with regular monthly meetings. Alcohol hand rub was introduced at each bed side of patient with subsequent improvement in the hand hygiene compliance to 50%. Regular infection control education to both physicians and staff were started in person and through tele-medicine. HAI surveillance for catheter associated urinary tract infection and surgical site infection was initiated. Antibiotic stewardship in the form of removing irrational combinations from the pharmacy and single dose surgical peri-operative antibiotic prophylaxis was initiated.

## Conclusion

With trained personnel & technology the Implementation of infection control program to rural community hospital is reality in India. The IC program implementation improves the IC knowledge, hand hygiene compliance and antibiotic prescriptions in the rural settings. In the future this model should be replicated to other smaller rural hospitals in India.

## Competing interests

None declared

